# Evaluation of the nutritional quality of French children breakfasts according to the Breakfast Quality Score (BQS)

**DOI:** 10.3389/fnut.2024.1430831

**Published:** 2024-07-03

**Authors:** Romane Poinsot, Sinead Hopkins, Matthieu Maillot

**Affiliations:** ^1^MS-Nutrition, Marseille, France; ^2^Cereal Partners Worldwide, Lausanne, Switzerland

**Keywords:** breakfast, meal, nutrient profiling, score, nutritional recommendations, nutritional adequacy, grains

## Abstract

**Background:**

Breakfast meals provide essential nutrients and energy to children and adolescents. Based on recommendations from the International Breakfast Research Initiative (IBRI), the Breakfast Quality Score (BQS) was developed to assess breakfast nutritional value among the French adult population. However, its application to children remains unexplored.

**Objective:**

This study aimed to evaluate the BQS in assessing the nutritional quality of breakfasts consumed by French children aged 4–17 years.

**Methods:**

A total of 4,015 breakfasts, obtained from dietary recalls provided by 1,448 children participating in the French representative dietary survey (INCA3), were analyzed. As for adults, the performance of the BQS was tested through correlations with other nutritional indicators and comparison of nutrients and dietary components between tertiles of scores. The study examined the regularity of breakfast consumption and compared the BQS of children’s breakfasts across socio-demographic modalities and breakfast categories distinguished by their primary grain component. Additionally, a predictive modeling using Partial Least Squares (PLS) regression identified influential INCA3 food groups.

**Results:**

The majority of children consumed solid breakfasts regularly. Socio-professional category, household income and food insecurity influenced BQS, revealing contradictory disparities. Ready-to-eat cereal (RTEC) breakfasts had the highest BQS (73.5% for children and 73.1% for adolescent), while biscuits and viennoiseries scored the lowest (52% for children and 49.1% for adolescent). PLS highlighted RTECs, milk, and hot beverages (mainly containing chocolate milk) as being positively associated with BQS, while sweet beverages and viennoiseries were negatively associated.

**Conclusion:**

The study demonstrates the effectiveness of the BQS in assessing children’s breakfast quality, with RTEC breakfasts showing the highest nutritional value. The findings provide insights into factors influencing breakfast nutritional quality and underscore the importance of promoting healthier breakfast choices among all children.

## 1 Introduction

Breakfast is commonly portrayed as the most important meal of the day, especially for children and adolescents, as it provides essential nutrients and energy necessary for their optimal growth, health, and cognitive functions (1–3). Although evidence from intervention studies regarding the effects of skipping breakfast on energy balance and body mass is inconclusive (1), several observational studies provide evidence that skipping breakfast could potentially contribute to weight gain and to the development of overweight and obesity (4, 5) during childhood and adolescence (5, 6). Additionally, there is evidence indicating that breakfast consumption can improve cognitive function, encompassing memory retention, academic achievement, and school attendance of children (3, 7, 8).

Despite variations in breakfast composition observed across studies and geographical regions, consistent evidence suggests that children who regularly consume breakfast exhibit a nutritionally more favorable diet compared to breakfast skippers (7, 9–13). Indeed, breakfast is a key contributor to daily energy and nutrient intakes among children (9, 10, 12, 13). In France, breakfast provides approximately 21% of children’s daily energy intake and > 21% daily intake of vitamins B and C, calcium, iron, iodine, manganese, phosphorus, potassium, and magnesium (14, 15). However, French breakfasts also contribute significantly to daily free sugar intakes (around 36%) (14). Moreover, children from lower socioeconomic groups are more prone to skipping breakfast than other children (3, 16), leading to poorer overall diet quality (3). While socioeconomic differences in breakfast consumption frequency have been extensively studied, there is a lack of research linking socioeconomic variables to breakfast meal quality.

Several studies have explored breakfast dietary patterns and their nutritional quality (10, 11, 17). In France, breakfasts containing Ready-to-eat cereals (RTECs) with milk (consumed by 18% of the studied children), tended to exhibit the most favorable nutritional profile (18). Breakfasts with fat or sweet foods (such as viennoiseries) (18), had the least favorable nutritional profile (10, 17).

Recently, the International Breakfast Research Initiative (IBRI) proposed a series of nutrient standards to assess the nutritional value of breakfast meals (19). The IBRI targets, developed for both adults and children, were based on actual consumption levels during breakfast and on overall nutrient adequacy in the US, Canada, France, Spain, the UK and Denmark (19). To simplify the extensive list of nutrient-based IBRI recommendations, an overall breakfast quality score (BQS) has been developed (20). The proposed BQS incorporates nutrients to limit as well as protein, fiber, and various essential micronutrients to encourage, offering a comprehensive assessment of breakfast quality. Although the BQS effectively ranks the nutritional density of adult breakfasts against IBRI recommendations (20), its application to children and adolescents remains untested.

The aim of the present study was to test the performance of the BQS in assessing the nutritional quality of breakfasts consumed by French children aged 4–17 years old and the association across socio-demographic modalities and breakfast categories characterized by the main grain component. Finally, a statistical model was developed to identify specific food groups associated with breakfast nutritional quality.

## 2 Materials and methods

### 2.1 Dietary data

#### 2.1.1 Survey description

Dietary information regarding breakfast consumption in France was obtained from the Third French Individual and National Food Consumption Survey (INCA3) study, which focuses on the dietary habits of the French population between 2014 and 2015. One single participant was randomly selected per household. A total of 2,698 children aged 0 to 17 years were recruited. Among them, 1,993 (67%) completed at least two dietary recalls out of three. The two or three non-consecutive 24-h dietary recalls collected included 2 weekdays and 1 weekend day. Children aged 0 to 14 years had a food diary, in which the child or any other person responsible for their diet (parents, grandparents, nanny, daycare or school staff, etc.) had to record the day’s food consumption. In contrast, adolescents aged 15 to 17 years were directly interviewed about their consumption from the previous day. Regardless of age, the interviews were conducted by telephone using the standardized software GloboDiet (formerly EPIC-Soft). The study protocol obtained approval from the National Commission for Data Protection and Liberties (CNIL) on May 2, 2013 (Decision DR 2013–228), following a favorable opinion from the Advisory Committee on Information Processing in Health Research (CCTIRS) on January 30, 2013 (opinion 13.055).

**Figure 1:**
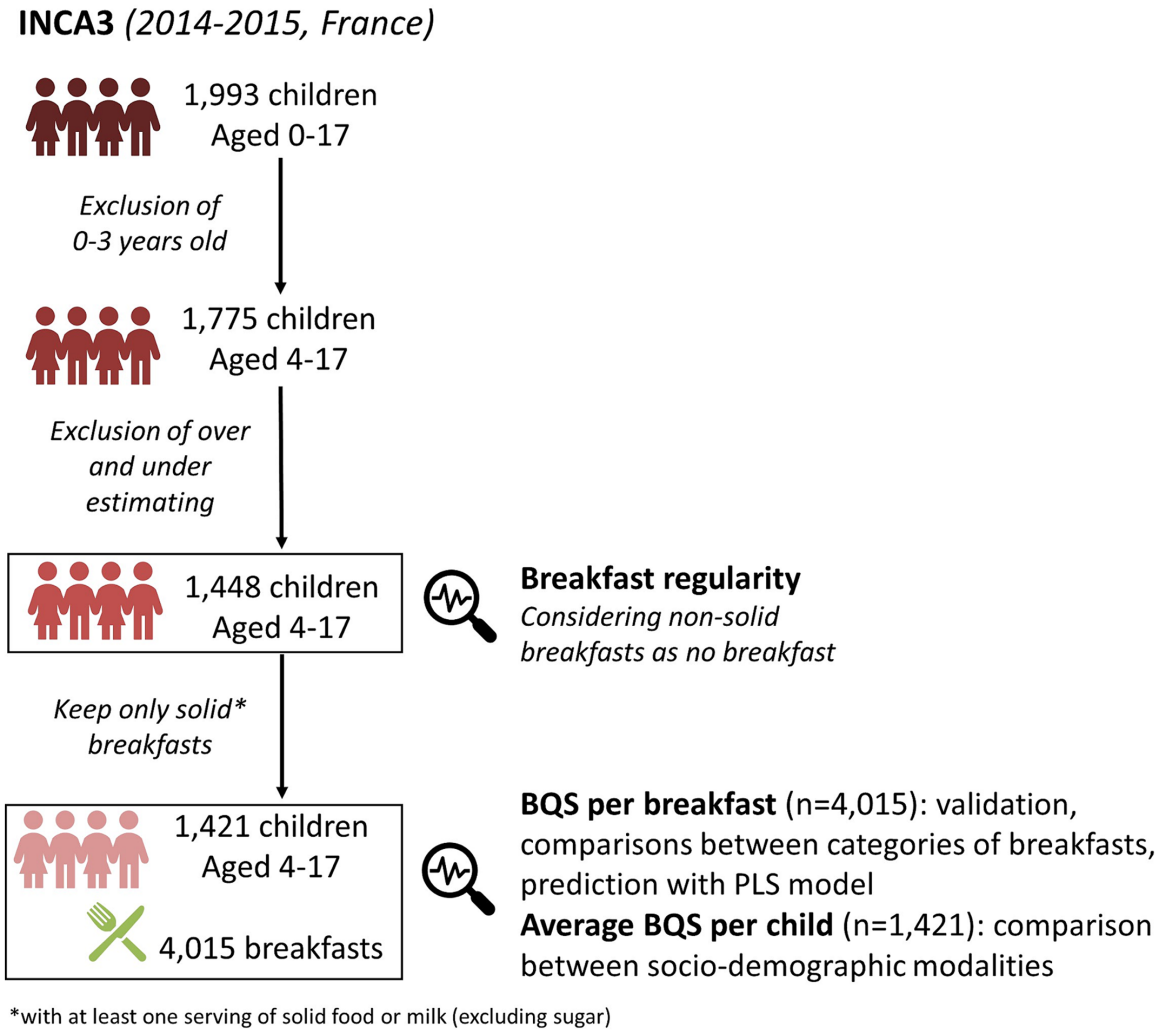
Overview of the study sample for the analyses on French children’s breakfasts. BQS, breakfast quality score.

The exact age of each child was not provided but children were divided into five age ranks: 0–3, 4–6, 7–10, 11–13, and 14–17 years old. Only children aged 4 and above were included in the study, totaling 1,775 participants. After exclusion of individuals for which total energy intakes were over-estimated and under estimated (provided in INCA3 data, according to adjusted physical activity level and Black’s equations), the final sample included 1,448 participants (Figure 1).

Nutrient composition data was obtained from the associated ANSES CIQUAL 2016 database, providing energy and nutrient values per 100 g of edible portion for all foods reported in the INCA3 study. Free sugars were derived from added sugars which were estimated following the Louie’s method (21). The foods reported as consumed are classified into 44 groups.

#### 2.1.2 Breakfasts data

During the dietary recall, participants were asked to specify the eating occasion, with 10 options available, including “breakfast.” Breakfast meal is composed by foods consumed during the identified breakfast occasion. Non-solid breakfasts, comprising solely of liquids such as hot beverages (with or without sweeteners) or soft drinks, were excluded. Milk was considered as solid. A total of 4,015 breakfasts consumed by 1,421 different individuals were included in the breakfast analysis (Figure 1).

#### 2.1.3 Socio-demographic characteristics

Household referent and participant socio-demographic variables were extracted from the INCA3 survey The socio-economic characteristics of the child and their reference person were collected face-to-face. The definition of the reference person used in the INCA3 study corresponds to that used by INSEE for the French population census (22). Household referent variables included socio-professional category (low, medium, high or inactive according to ISCO nomenclature), household monthly income per consumption unit (<900 €; [900–1,340] €; [1340–1850]€; ≥1850 €; No answer), education level (Primary and middle schools; Highschool; Undergraduate studies; Graduate studies), and food insecurity level (Food Secure; Moderate food insecurity; Severe food insecurity). A shortened 6-question version of the HFSSM tool was used to estimate the food insecurity level. Households are considered food secure for a score of 0 or 1, moderately food insecure for a score between 2 and 4, and severely food insecure for a score of 5 or 6.

Participant socio-demographic variables included sex, age range (4–6 y, 7–10 y; 11–14 y; 15–17 y), physical activity level (low; medium; high corresponding, as much as possible and according to available data, to the recommendation of the World Health Organization), BMI classes (Thinness; Normal; Overweight and obesity according to the international definition established by the Childhood Obesity Working Group of the International Obesity Task Force and specific to age and sex), season, and total screen time (≤ 0.5 h/day; 0.5–1 h/day; > 1 h/day). Total screen time was calculated as the sum of TV, video game, and computer times. Screen time ranges were chosen arbitrarily.

#### 2.1.4 Categorization of breakfasts

The 4,015 solid breakfasts were categorized based on the four types of grain-based foods contributing the most to the energy content of the breakfast. If a breakfast contained no grains (e.g., only dairy products, fruits, or confectionery), it was categorized as a non-grain solid breakfast. This categorization resulted in five different categories: (1) whole grain breads, (2) refined breads, (3) RTECs (both refined and wholegrain), (4) biscuits and viennoiseries, and (5) non-grain.

### 2.2 Regularity of breakfast consumption

The study sample of children was described based on their reported frequency of breakfast consumption. Among the 1,448 participants aged 4–17 included in the analysis (Figure 1), the number and percentage of breakfast skippers, as well as the percentage of participants consuming 1, 2, or 3 solid breakfasts, were determined. Non-solid breakfasts were considered as non-breakfasts. Percentages were weighted using individual weighting factors provided in the INCA3 survey. Chi-squared tests were also applied to compare these percentages regarding age ranges and sex.

### 2.3 Estimation of breakfast quality score (BQS) in French children

#### 2.3.1 Description of BQS

The BQS has been described in detail elsewhere (20). The BQS is composed of three subscores derived from the weighted arithmetic mean of corresponding nutrient adequacy: *n* subscore called “eLIMf” based on energy, saturated fat, free sugars, and sodium adequacies, a subscore called “PF” based on protein and fiber adequacies, and a subscore called “VM” based on 14 vitamins and minerals adequacies. The three subscores eLIMf, PF and VM account for 50, 43.75, and 6.25% of the BQS, respectively. The BQS ranges from 0 to 100, 100 meaning that the breakfast is compliant with all IBRI recommendations. Nutrient adequacies used in the present study were the breakfast nutrient recommendations for children previously published by the IBRI consortium (19). The recommendations are the same regardless of child age. Nutrient standards for vitamins B1, B2, B12, and calcium were higher for children than those for adults.

**Figure 2:**
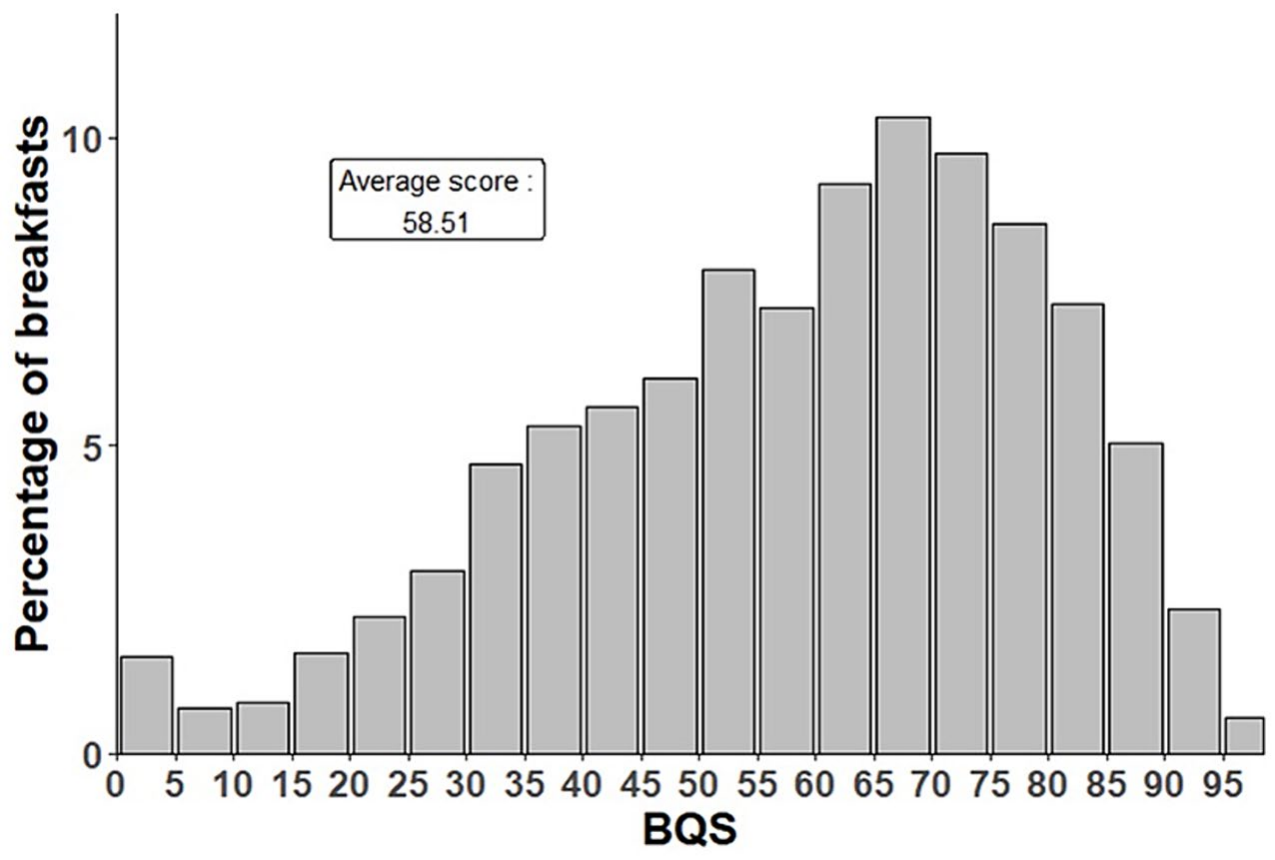
BQS distribution among solid breakfasts consumed by children aged 4–17 in INCA3 (*n* = 4,015).

The BQS was estimated on the 4,015 solid breakfasts (Figure 2).

#### 2.3.2 Validation of BQS among children

The BQS for the 4,015 breakfast meals were compared to the nutrient-rich food score (NRF 9.3), LIM (from SAIN, LIM score) and energy content calculated for the same breakfast meals.

The NRF9.3 nutrient profiling model is the sum of percent daily values (DV) for nine nutrients to encourage (proteins, fibers, calcium, iron, magnesium, potassium, vitamin C, vitamin A, and vitamin D) minus the sum of percent maximum recommended values (MRV) for three nutrients to limit (saturated fats, added sugars, and sodium) (23). All values are calculated per 100 kcal and the ratio for nutrients to encourage were capped at 100%. NRF was calculated using the following equation:


$$NRF=\left(\overset{9}{\underset{i=1}{\sum }}\frac{\frac{\mathrm{Intak}e_{i}}{\mathrm{Energy}}\times 100}{DV_{i}}-\overset{3}{\underset{j=1}{\sum }}\frac{\frac{\mathrm{Intak}e_{j}}{\mathrm{Energy}}\times 100}{MRV_{j}}\right)\times 100$$

The LIM score is the negative subscore of SAIN, LIM score. It represents the percentage of excess compared to the maximum recommended values of nutrients to be limited (saturated fatty acids, free sugars and sodium) (24). It is calculated for 100 g of food, using the following equation (Weight equates to grams of the breakfast meal):


$$LIM=\frac{\frac{\frac{\mathrm{Intak}e_{Na}}{\mathrm{Weight}}\times 100}{3153}+\frac{\frac{\mathrm{Intak}e_{SFA}}{\mathrm{Weight}}\times 100}{22}+\frac{\frac{\mathrm{Intak}e_{\mathrm{Free}\;\mathrm{sugars}}}{\mathrm{Weight}}\times 100}{50}}{3}\times 100$$

Correlations between BQS and the three indicators were estimated using the Spearman rank-order correlation coefficient. The BQS performs well for children if it has a positive correlation with the NRF and a negative correlation with the LIM.

BQS values for the 4,015 breakfasts were also split into tertiles. The general linear model then compared values of energy and nutrients (for which IBRI recommendations were available), subscores eLIMf, PF, and VM, and grams of nine dietary components across the BQS tertiles. The nine dietary components of interest were fruits and vegetables, RTECs, whole-grain foods (excluding RTECs), refined-grain cereals (excluding RTECs), milk and dairy, plant fats, animal fats, sugary foods, and sweet-tasting beverages (e.g., soda and fruit juices). A *post-hoc* comparison (Tukey’s HSD test) was performed when the difference was significant.

#### 2.3.3 Comparison of BQS values

##### 2.3.3.1 Comparison of average BQS according to socio demographic characteristic

The BQS was analyzed in relation to participant and household reference socio-demographic variables. The statistical analysis has been conducted at the participant level which means that the BQS corresponded to the average BQS of a participants’ breakfasts over the 2 or 3 recalls. When a breakfast was skipped, it was not assigned a value of 0 but rather excluded from the mean computation. To ensure sample representativeness, all analyses at participants levels accounted for the INCA 3 sampling frame design and were weighted for unequal sampling probabilities and differential non-responses by gender, age, size of the household, season, region, size of urban area and occupation and SPC of the reference person for the household. Additionally, chi-square tests were conducted, adjusting for the INCA3 sampling frame design, to examine potential significant associations between socio-demographic characteristics and breakfast categories.

##### 2.3.3.2 Mapping of breakfast categories according to the energy and BQS

Food group intakes, as well as BQS and its three subscores, were compared across the five breakfast categories. Scores comparisons were done among young children’s (4–10 years old) breakfasts and among adolescent’s (11–17 years old) breakfasts separately. The statistical analysis has been conducted at the breakfast level. The general linear model compared INCA3 food groups intakes, BQS, PM, VM and eLIMf across the breakfast categories. A *post-hoc* comparison (Tukey’s HSD test) was performed when the difference was significant. Only food groups with a reported intake >1 g at breakfasts were presented. Moreover, average BQS per categorized breakfast was plotted against energy intake.

Statistical analyses used the R software version 4.3 and SAS 9.4 software for the tests with the sampling frame design (PROC SURVEY FREQ). The level of significance was set to 5% for all tests.

#### 2.3.4 Prediction of BQS value

The Partial Least Squares (PLS) regression modeling was employed to predict the BQS based on INCA 3 food groups variables. PLS regression is a statistical technique used for modeling the relationships between a set of independent variables (predictors) and a dependent variable (response). In the present study, the food group variables served as predictors, representing the dietary composition of breakfasts consumed by the children. The BQS, which reflects the overall nutritional quality of breakfasts, was the dependent variable of interest. Applying PLS regression aimed to identify the food groups with the strongest associations to the nutritional quality of children breakfast. PLS model was preferred to multiple linear regression in order to account for the multicollinearity of food group composition of breakfast [e.g., milk (or butter)] can be consumed more frequently with RTEC (or bread).

Model fit statistics, such as R^2^ were evaluated to determine the overall performance of the model. In this analysis, R^2^ represents the proportion of variance in the BQS explained by the food groups intakes. The higher the R^2^, the better the fit of the model to the data. The beta coefficients of the predictor variables, which indicate the strength and direction of food group’s influence on the BQS were also analyzed. Additionally, the variable importance in projection (VIP) scores were utilized to identify the most influential predictor variables (25). While VIP cut-off thresholds differ across studies, conventionally, variables with VIP scores below 0.8 are considered to have minimal influence in the model.

## 3 Results

### 3.1 Regularity of breakfast consumptions

The percentage of French children in the study sample consuming 1, 2 or 3 breakfasts over 2 or 3 recalls is detailed in Supplementary Table S1. Among 1,448 French children, most (83%) consumed a solid breakfast at every morning of their recall. Only 3% (*n* = 27) skipped it during every recall, and 13% consumed it 1 or 2 times out of 3 recalls. Sex did not influence the regularity of breakfast intake, but age range did with younger children tending to consume breakfast more regularly (91.3%) than older adolescents (64.6%).

### 3.2 Validation of the BQS among children

#### 3.2.1 Score distribution

In the study sample, there were 4,015 solid breakfasts consumed among children aged 4 to 17 years. The distribution of their BQS values is shown in Figure 2. BQS ranged from 0 (2% of the breakfasts) to 96 out of 100, with a mean of 58.5%. A score of 0% indicates that the breakfast resulted in more negative points (from the eLIMf subscore) than positive points from PF and VM. The BQS distribution exhibits a rather normal shape, although slightly negatively skewed.

#### 3.2.2 Correlations with energy and nutritional quality indicators

Spearman correlations between BQS and nutritional indicators and between BQS and energy were significant. BQS was negatively correlated to the score of nutrients to be limited LIM (*r* = −0.26, *p* < 0.001) and positively correlated with NRF9.3 nutrient density score (*r* = 0.53, *p* < 0.001, Supplementary Figure S1). Spearman correlation between BQS and energy was low (*r* = 0.089, *p* < 0.001).

#### 3.2.3 Comparison of nutrients and dietary components contents between tertiles of BQS

BQS varies from 0 to 50.9% in the first tertile of BQS and from 70.1 to 98.7% in the third tertile. Table 1 shows that as BQS values increased, the eLIMf subscore along with the PF and VM subscores also increased. Notably, except for sodium, the amounts of nutrients to be limited decreased between the low and medium tertiles, and between the medium and high tertiles of BQS. Conversely, with the exception of fiber and vitamin C, the amounts of nutrients to be encouraged increased between the low and high tertiles and between the medium and high tertiles of BQS. There was no significant difference in energy content between the low and medium tertiles, but it was significantly lower in the high tertiles. Additionally, intakes of food groups such as fruits, vegetables, whole-grain foods, RTEC, milk and dairy products, and plant-based fats were highest in the breakfasts in the top tertile of BQSwhile intakes of refined-grained foods, animal fats, sugary foods, and sweet-tasting beverages were the lowest in breakfasts in the top tertile.

**Table 1:** Mean BQS, subscores, nutrient, and dietary component intakes in all breakfasts and across breakfasts in the three tertiles of BQS.

	All [0;98.7]		Low tertile [0;50.8]		Medium tertile [50.9;70.1]		High tertile [70.1;98.7]		*p*-value
	Mean	SD	Mean	SD	Mean	SD	Mean	SD	
BQS	**58.5**	20.8	**34.3**	12.9	**61.2**	5.57	**80.0**	6.57	***
Subscore eLIMf	**23.3**	16.3	**7.88**	15.4	**24.7**	8.90	**37.4**	6.42	***
Energy (kcal)	422	223	431^a^	305	439^a^	197	397^b^	125	***
SFA (% EBI)	14.0	6.84	15.3	8.79	14.3	6.46	12.5	4.13	***
Free sugars (% EBI)	20.3	12.4	28.6	13.9	18.8	10.1	13.5	7.05	***
Sodium	369	287	366^a^	401	392^b^	245	348^a^	160	***
Subscore PF	**4.30**	1.41	**3.49**	1.69	**4.58**	4.16	**4.83**	0.84	***
Proteins (g)	12.6	7.54	8.97	8.67	13.1	6.78	15.6	5.23	***
Fibers (g)	3.03	2.19	2.91	2.50	3.14	2.18	3.04	1.83	0.023
Subscore VM	**30.8**	10.3	**22.7**	10.7	**31.9**	7.85	**37.8**	4.71	***
Calcium (mg)	314	234	160	203	321	208	461	184	***
Iron (mg)	2.48	1.89	1.90	1.90	2.43	1.87	3.11	1.69	***
Magnesium (mg)	66.0	39.5	52.8	46.9	68.0	37.3	77.1	28.0	***
Potassium (mg)	653	417	526	490	668	403	765	299	***
Zinc (mg)	1.42	0.88	0.94	0.92	1.47	0.81	1.84	0.66	***
Vitamin A (μg RAE)	110	149	89.8	139	113	133	128	170	***
Vitamin B1 (mg)	0.39	0.29	0.25	0.26	0.39	0.29	0.51	0.27	***
Vitamin B2 (mg)	0.62	0.47	0.32	0.39	0.63	0.42	0.91	0.41	***
Vitamin B3 (mg)	2.81	3.18	1.78	2.43	2.69	3.29	3.97	3.34	***
Vitamin B6 (mg)	0.38	0.35	0.25	0.29	0.37	0.35	0.51	0.34	***
Vitamin B9 (mg)	80.1	54.0	68.6	57.1	80.2	55.7	91.4	45.9	***
Vitamin B12 (μg)	1.15	0.93	0.59	0.82	1.16	0.82	1.71	0.79	***
Vitamin C (mg)	27.5	32.3	28.0	34.0	27.5	34.7	27.0	27.7	0.728
Vitamin D (μg)	0.84	0.84	0.42	0.64	0.89	0.84	1.21	0.81	***
Dietary components									
Fruits and vegetables (g)	8.34	35.0	5.29^a^	24.3	8.62^b^	33.2	11.1^b^	44.3	***
RTECs (g)	11.71	24.0	3.23	14.7	10.4	24.9	21.5	27.1	***
Whole-grain cereals (g)	2.93	14.8	2.29	13.5	3.42	17.1	3.07	13.7	***
Refined-grain cereals (g)	17.2	38.4	21.7^a^	51.2	19.4^a^	34.8	10.7^b^	22.9	***
Milk and dairy products (g)	176	163	64	127	182	143	281	141	***
Plant fats (g)	0.56	3.36	0.42	3.21	0.68	3.75	0.58	3.08	0.132
Animal fats (g)	1.86	5.37	2.59	7.02	2.08	5.21	0.91	2.94	***
Sugary foods (g)^1^	54.4	54.2	63.3	61.1	57.5	54.4	42.3	42.8	***
Sweet-tasting beverages (g)^2^	72.5	109	110	128	73.7	108	33.7	67.6	***

***p < 0.001. ^1^Sugary foods included biscuits, cakes, pastries, viennoiseries, chocolate, jam and honey. ^2^Sweet-tasting beverages included regular and sugar-free sodas, plant-based beverages as well as fruit juices. Same index letters (e.g., a and a) indicates that there is no significant difference between the two tertiles and different index letters (e.g., a and b) indicate that the difference is statistically significant between the two tertiles. Bold values are BQS and subscores values.

### 3.3 Description of French children’s breakfast through BQS

#### 3.3.1 Comparison of BQS between socio-demo modalities

There were 1,421 children aged 4–17 in the study sample who consumed at least one breakfast. The average BQS of their breakfasts, by demographic categories, is shown in Table 2. BQS did not differ significantly between the sex, age range, physical activity levels, screen time, BMI classes or season. Regarding socio-demographic factors related to the household, socio-professional category, income per consumption unit, and food insecurity level influenced BQS. Higher socio-professional categories were associated with higher average BQS scores. However, children whose household referent was inactive tended to have a better BQS. Additionally, BQS was significantly higher in the lowest income group (61.30 for income <900€ per CU) compared to the highest income group (56.32 for income ≥1,850€ per CU). Likewise, the average BQS for children in households experiencing food security was 58.83, while it was 66.79 in households experiencing severe food insecurity. BQS values did not significantly differ across levels of education.

**Table 2:** BQS of children according to socio-demographic modalities and household referent.

Variable	Modality	*N*	BQS		*p*-value
			Means	SE	
Socio-professional category^2^ (household referent)	Low	438	57.54	1.03	0.0332
	Medium	492	59.51	0.91	
	High	442	60.11	1.02	
	Inactive^1^	48	63.34	2.22	
Household monthly income per consumption unit (household referent)	<900 €	281	61.30	1.39	0.0378
	[900–1,340]€	365	59.49	1.14	
	[1,340–1,850]€	412	57.65	1.21	
	≥1850 €	256	56.32	1.34	
	No answer	107	61.63	2.10	
Education level (household referent)^3^	Primary and middle schools	437	58.03	1.05	0.3314
	Highschool	300	60.42	1.53	
	Undergraduate studies	339	58.35	1.00	
	Graduate studies	336	60.02	1.03	
Sex (participant)	Male	720	59.12	0.81	0.9630
	Female	701	59.06	0.91	
Age range (participant)	4–6 years old	324	59.13	1.22	0.5466
	7–10 years old	447	60.19	0.94	
	11–14 years old	410	58.39	1.18	
	15–17 years old	240	57.62	1.93	
Food insecurity level (household referent)	Food Secure	1,319	58.83	0.64	0.0100
	Moderate food insecurity	77	60.17	4.66	
	Severe food insecurity	25	66.79	2.57	
Physical activity level (participant)^4^	Low	551	59.85	1.05	0.2379
	Medium	536	57.65	1.02	
	High	333	60.13	1.11	
Total Screen time^5^ (participant)	Half an hour or less per day	1,028	59.44	0.75	0.3728
	Between 0.5 and 1 h per day	304	57.51	1.30	
	More than 1 h per day	84	59.98	2.51	
BMI classes (participant)	Thinness	167	58.49	1.96	0.8423
	Normal	1,057	59.30	0.66	
	Overweight and obesity	197	58.45	1.44	
Season	Winter	492	60.47	1.13	0.4607
	Spring	431	57.43	1.32	
	Summer	252	59.22	1.15	
	Autumn	239	59.23	1.51	

^1^Unemployed and retired; ^2^Modality is not available for 1 participant; ^3^Modality is not available for 9 participants; ^4^Modality is not available for 1 participant; ^5^Modality is not available for 5 participants.

#### 3.3.2 Nutritional quality of categorized French children breakfasts

Breakfasts were categorized into 5 groups based on the primary source of grain-based foods. Table 3 displays the average intake of INCA3 food groups in the categorized breakfasts. The biscuit and viennoiserie category is the most prevalent (*n* = 1,558), consisting mainly of viennoiseries (65 g) and fruit juice (69 g). The refined bread category (*n* = 1,078) closely resembles the traditional French breakfast, consisting of refined bread associated with animal fats (5.18 g on average), jam (8.21 g), fruit juice (72 g), and hot chocolate milk (158 g) or plain milk (54.9 g). The wholegrain bread category is less common (*n* = 127) but similar to the refined bread category, but with wholegrain bread replacing refined breads. The RTECs category (*n* = 912) is characterized by an average consumption of 50 g of RTECs, typically paired with plain milk (216 g) and fruit juice (57 g). Finally, solid breakfasts without any grain components (*n* = 340) are characterized by the consumption of fresh and dried fruits or cooked fruits like compote, hot chocolate milk (182 g), and yogurts (18 g). Salty foods are very lightly represented in French children breakfasts, with a maximum of only 1.28 grams of cheese on average, in the refined breads category.

**Table 3:** INCA3 Mean food group intakes in categorized French children’s breakfasts.

Food group intake (g/breakfast)	All breakfasts *N* = 4,015		Biscuits & viennoiseries *N* = 1,558		Refined bread *N* = 1,078		Wholegrain bread *N* = 127		RTECs *N* = 912		Non-grain *N* = 340		*p* value
	Mean	Std	Mean	Std	Mean	Std	Mean	Std	Mean	Std	Mean	Std	
Refined breads	18.1	38.8	1.15^a^	6.9	63.6^b^	51.1	0.08^a^	0.93	2.36^a^	10.4	0^a^	0	<0.0001
Animal fats (butter)	1.85	5.37	0.53 ^b^	2.97	5.18 ^a^	8.06	5.31^a^	8.29	0.36 ^b^	2.05	0.04 ^b^	0.56	<0.0001
Vegetable fats (oils)	0.56	3.36	0.21 ^b^	2.04	1.34 ^a^	5.17	2.43 ^a^	6.87	0.13 ^b^	1.61	0.1 ^b^	0.89	<0.0001
Whole grains breads	1.78	11.9	0.16 ^b^	3.42	0.05 ^b^	1.23	53.2 ^a^	40.0	0.07 ^b^	1.19	0 ^b^	0	<0.0001
Fruits (fresh and dried)	5.49	28.9	4.46 ^b^	25.4	4.88 ^b^	26.3	7.83 ^b^	28.2	5.54 ^b^	26.3	11.1 ^a^	50.3	0.0028
Compote	3.06	20.1	3.05 ^a^	18.2	1.74 ^a^	14.8	2.13^a,b^	13.7	3.06 ^a^	23.9	7.6 ^b^	30.8	0.0002
Chocolate products	5.81	15.6	6.3^b^	16.7	10.2^a^	19.1	9.91^a,b^	20.8	0.58^c^	3.31	2.18^c^	10.2	<0.0001
Sugar, jam and honey	4.04	11.7	2.59^c^	9.11	8.21^b^	16.2	13.2^a^	19.9	1.34^c^	6.6	1.3^c^	4.72	<0.0001
Bottled water	7.7	40.2	9.82^a,b^	45.1	5.7^a^	33.2	4.63^a^	31.2	8.84^a^	45.2	2.69^a,c^	19.6	0.0081
RTECs	11.7	24.0	1.22^a^	6.75	1.36^a^	7.35	0.13^a^	1.34	47.84^b^	26.7	0^a^	0	<0.0001
Tap water	12.8	52.9	12.^8^	50.8	15.3	60.8	9.12	37	12.26	53.0	7.52	37.5	0.1612
Soft drinks, syrups, fruit nectars, flavored waters	7.81	44.0	9.58	47.3	6.2	34.4	9.8	71.6	7.13	45.0	5.87	38.4	0.2753
Fruit juices	63.3	104	69.2^a^	106	72.1^a^	109	56.7^a,c^	102	56.68^a,d^	98.2	29.1^c^	75.0	<0.0001
Hot drinks (e.g., chocolate milk)	119	178	107^c^	168	158^b^	197	150^a,b^	167	65.5^a,c^	149	182^b^	182	<0.0001
French viennoiseries, biscuits & pastries	25.7	39.9	64.9^b^	39.3	0.82^a^	5.77	0.65^a^	4.76	1.3^a^	7.59	0^a^	0	<0.0001
Milk	90.8	162	54.7^a^	128	54.9^a^	135	66.2^a^	136	216^b^	194	43.7^a^	117	<0.0001
Yogurts	9.27	36.9	9.5^a^	38.2	8.88^a^	36.0	5.43^a^	24.1	6.53^a^	32.8	18.2^b^	45.5	<0.0001
Cheese	0.47	4.47	0.14^a^	2.02	1.28^b^	7.58	0.97^a^	6.27	0.1^a^	1.67	0.29^a^	3.21	<0.0001

Only food groups with a mean intake of at least 1 g in one or more category are represented. Same index letters (e.g., a and a) indicates that there is no significant difference between the two categories and different index letters (e.g., a and b) indicate that the difference is statistically significant between the two categories.

**Figure 3:**
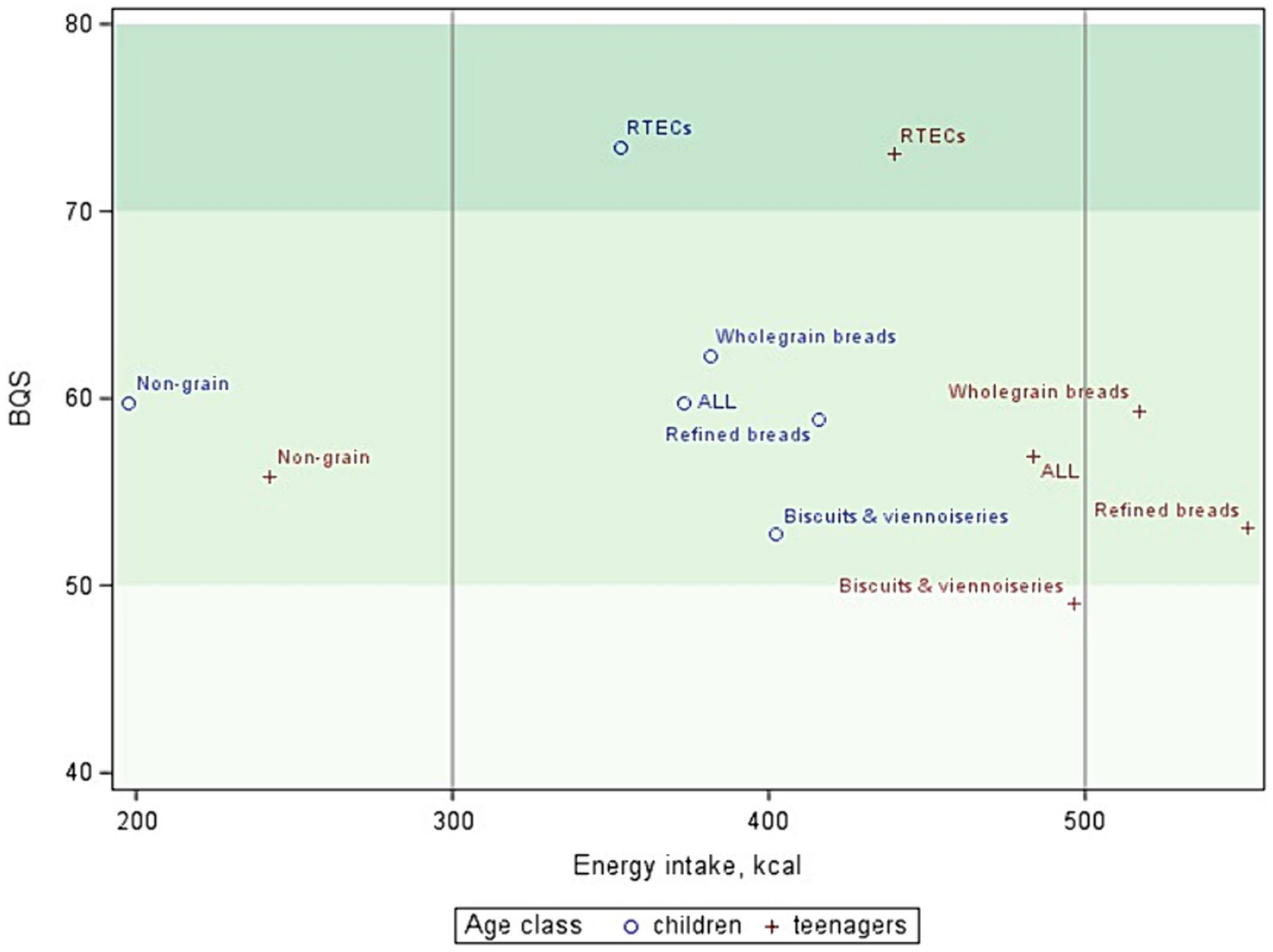
Average BQS of breakfast categories as a function of energy, for children and adolescents. Color bands represent the three tertile levels.

The average BQS and energy content for each breakfast category were calculated among two age classes: young children (4 to 10 years old) and adolescents (11 to 17 years old). These data are illustrated on a two-dimensional map in Figure 3, with each tertile of scores depicted in a different color. RTECs breakfasts displayed the highest BQS, with all falling within the highest tertile, averaging around 73 (Table 4). Conversely, the biscuits and viennoiseries category had the lowest average BQS, reaching around 50 regardless of age class. Adolescents tended to consume higher energy breakfasts (above the recommended 500 kcal based on a 2,000 kcal diet for bread categories) compared to children. On the contrary, non-grain breakfasts were insufficient in energy, falling below 300 kcal (Figure 3).

**Table 4:** Comparison of BQS and sub scores of BQS between breakfast categories.

		*N*	BQS	SD	PF	SD	VM	SD	eLIMf	SD
Theoretical ranges			0–100		0–6.2		0–43.8		0–50	
Age class	Category									
Children	Wholegrain bread	72	62.3^b^	15.7	4.77^a^	1.30	29.6^b,c^	9.33	28.0^a,b^	12.7
	Refined bread	575	59.0^b^	18.9	4.39^a^	1.41	29.2^b^	9.55	25.4^b^	15.0
	RTECs	467	73.5^a^	15.4	4.31^a^	1.21	37.6^a^	6.03	31.6^a^	12.0
	Biscuits & viennoiseries	871	52.8^c^	19.2	3.97^b^	1.32	27.0^c,d^	9.98	21.7^c^	14.3
	Non-grain	237	59.8^b^	20.9	3.32^c^	1.22	25.9^d^	8.81	30.4^a^	15.9
	*p*-values		***		***		***		***	
Adolescents	Wholegrain bread	55	59.3^b^	19.8	5.48^a^	1.35	32.1^b^	9.77	21.5^b,c^	16.3
	Refined bread	503	53.1^b^	20.3	4.83^b^	1.42	31.7^b^	9.86	16.5^c^	19.8
	RTECs	445	73.1^a^	16.8	4.68^b^	1.17	39.8^a^	5.43	28.6^a^	14.5
	Biscuits & viennoiseries	687	49.1^c^	19.1	4.32^c^	1.47	28.2^c^	10.8	16.6^c^	15.3
	Non-grain	103	55.9^b^	25.9	3.39^d^	1.43	27.3^c^	10.1	24.9^b^	19.5
	*p*-values		***		***		***		***	

***p < 0.001. Same index letters (e.g., a and a) indicates that there is no significant difference between the two categories and different index letters (e.g., a and b) indicate that the difference is statistically significant between the two categories.

The highest BQS for the RTECs breakfast category is explained by a much higher VM subscore, as well as a higher eLIMf subscore (Table 4), indicating lower levels of saturated fats, free sugars, and/or sodium compared to other categories. On the other hand, the lowest BQS of the biscuits and viennoiseries category is explained by its low eLIMf subscore. Moreover, RTECs breakfasts showed the highest amount of milk, whereas refined and whole grains are characterized by a higher amount of animal fats (mostly butter) and sweetening ingredients such as jam, honey, or sugar. Non-grain breakfasts had the lowest PF and VM subscores but the highest eLIMf subscores, resulting in an intermediate BQS value. The number of wholegrain bread breakfasts may not be sufficient to detect significant differences with refined bread breakfasts.

### 3.4 Predicting the BQS value with food groups

The final PLS regression model comprised four PLS components (determined through cross-validation). This model accounted for 45% of the total variability in BQS. The PLS regression highlighted the food groups which were positively or negatively associated to the BQS. Food groups with a significant impact on BQS were identified based on a VIP coefficient exceeding 0.8 in Table 5. These influential food groups included chocolate products, RTECs, Sweet beverages, Fruit juices, Hot drinks (e.g., chocolate milk), French viennoiseries, biscuits & pastries, and Milk. The beta coefficients reflect the weights assigned to each food group in predicting BQS. For instance, the presence of Hot beverages and Milk in a breakfast increased the BQS by 21 and 17 points, respectively. Additionally, RTECS had a positive effect, estimated at 12 points. Viennoiseries, sweet beverages, and fruit juices were negatively associated with BQS (−3.9, −11.8, and −5, respectively). While Fruits and compote exhibited high beta coefficients (approximately +10), they were considered as non-significant in predicting the BQS, possibly due to their low level of reported consumption.

**Table 5:** Variable importance in projection (VIP) and beta coefficients of the Partial Least Squares regression model for each food group.

Food groups	Percentage of consumptions	VIP	beta
Intercept			45.86
Hot drinks (e.g., chocolate milk)	37.88	1.89	21.34
Milk	31.71	1.75	17.19
RTECs	25.73	2.1	12.2
Fruits (fresh and dried)	5.45	0.44	10.77
Compote	2.81	0.28	10.44
Yogurts	7.07	0.5	7.26
Vegetable fats (oils)	4.26	0.29	4.75
Whole grains breads	3.41	0.17	3.57
Cheese	1.47	0.12	2.67
Refined breads	29.89	0.51	2.25
Chocolate products	20.97	0.88	0.05
Bottle water	4.76	0.24	−1.92
Animal fats (butter)	16.11	0.46	−2.62
Tap water	7.55	0.35	−3.3
French viennoiseries. Biscuits & pastries	40.4	1.55	−3.88
Fruit juices	34.99	1.1	−5.01
Sugary foods (e.g., marmelade)	18.88	0.76	−10.09
Sweet beverages	4.11	0.82	−11.76

## 4 Discussion

The findings presented in this study highlight several key aspects regarding the nutritional quality of breakfasts consumed by French children and adolescents. Firstly, the analysis revealed that the majority of French children consistently consumed solid breakfasts, with only a small percentage (3%) skipping breakfast altogether. The estimation of the BQS provided further information on nutritional quality of those breakfast meals consumed. “RTECs” breakfasts emerged as the category with the highest BQS, characterized by higher levels of essential vitamins and minerals and a better eLIMf subscore. Conversely, “biscuits and viennoiseries” breakfasts which were the most frequently consumed by children and adolescents exhibited the lowest BQS, largely due to their higher content of nutrients to be limited. The BQS highlighted some contradictory disparities in breakfast quality based on socio-economic status. Lastly, our predictive modeling showed that specific foods including hot drinks (mainly hot chocolate milk), RTECs, and milk were significant predictors of a higher BQS while other foods including sweet beverages, fruits juices and French viennoiseries were predictors of a lower BQS.

Similar to the previously published data for adults (20), the distribution of BQS values exhibited a normal shape. Spearman correlation (*r* = 0.53) with NRF was also close to the correlation among adults (*r* = 0.55) (20). Comparisons between tertiles also yielded significant results, indicating that the amount of nutrients and foods to encourage increased with a higher BQS while the amount of nutrients and foods to be limited decreased. This validates the reliability of the BQS for children. Vitamin C was the only nutrient that did not significantly change between tertiles. Considering that only about 5% of individuals consume whole fruits, we can assume that a significant portion of vitamin C intake originates from fruit juices. However, it’s worth noting that fruit juices also contribute to free sugar intakes. Since free sugars negatively impacts the BQS, breakfast options featuring fruit juices may receive low scores despite offering vitamin C. Despite an overall low fiber intake across tertiles (around 3 g per breakfast), the BQS was able to slightly discriminate fiber intake. It is plausible that the fiber content of some products has improved since this survey due to reformulation efforts and that the discrimination of breakfasts based on fiber content would be better using more recent food consumption data (26).

The categorization of breakfasts into distinct categories based on grain-based food sources provided additional insights into breakfast patterns of French children and how they influence the BQS value. Breakfasts primarily composed of RTECs showed the highest BQS. The favorable BQS of “RTECs” breakfasts could be attributed to the content of fiber and micronutrients, as well as the co-consumption with milk, which was highest in RTECs breakfasts. Previous studies have demonstrated the good nutritional quality of breakfasts containing RTECs (18, 27–29). In France, ‘RTEC + milk’ pattern identified through PCA among children aged 9–11 has been previously described as the most advantageous, according to the diet quality indicator Mean Adequacy Ratio adjusted for energy (18). Some studies has already shown that at breakfast, cereal consumers consumed significantly higher fiber, calcium, iron, folate, total sugars and carbohydrate, but significantly lower total fat and sodium than non-cereal consumers (11, 27, 29, 30). Although it is not evident through the subscore of nutrients to be limited (eLIMf) in the present study, other studies have reported an association between frequent consumption of RTECs with daily total sugar intake (29) or total sugar intake at breakfast (28). Conversely, non-grain breakfasts had a lower energy and nutrient density with lowest protein/fiber and vitamins/minerals subscores which can be explained by the lack of a grain component. These breakfasts also had a better eLIMf subscore than most others which could be, due to lower fruit juices intake than in the other categories of breakfasts (11). Our study demonstrates that the type of grain-based food affected the BQS, with “Biscuits & viennoiseries” and “refined breads” having the lowest scores. However, the low number of breakfasts with “wholegrain bread,” as well as the inability to distinguish between whole and refined grain RTECs in our data, prevented us from confirming the superior nutritional quality of whole grains demonstrated in previous studies (7, 14).

Results from the PLS model were consistent with the comparison of breakfast categories and might explain what constitutes a good breakfast. Foods associated with a high nutritional quality were milk (including hot chocolate), RTECs, fruits, compote and yogurt, while those associated with a lower nutritional quality were viennoiseries and soft drinks (including fruits juices). With the highest VIP, RTECs were the most influential predictor. The positive effect of fruits, compote and yogurts on a higher nutritional quality was not considered as significant due to a low VIP (<0.8) but it can be explained by a low percentage of consumers (<7%). The average score indicates that children’s breakfasts were of higher nutritional quality (58.51), on average, compared to those of adults (51.62) (20). Children and adolescents consume more RTEC breakfasts than adults which might be one explanation why their BQS is higher.

The French National Nutrition and Health Program (PNNS) recommends to include four food components at breakfast: one cereal product such as whole grain bread or unsweetened cereal, one dairy product, one whole fruit or fruit juice and one “not too sweet” beverage (coffee, tea, water) (31). However, the PNNS consider RTECs as sweet products which should not be consumed often. In contrast, our findings show that RTEC regardless of their sugar content are associated with a higher breakfast quality score. Notably the RTEC breakfast was associated with the highest intake of milk and had one of the lowest intake of chocolate products, sugar, jam & honey and viennoiseries which may explain the better eLIMf score of these breakfasts.

The present study also delved into the comparison of BQS across different socio-demographic modalities. Household socio-professional category, income per consumption unit, and food insecurity level were the only variables that had significant effects on BQS. Children from households where the reference person was inactive (unemployed or retired), in the lowest income group, or experiencing severe food insecurity tended to have a better BQS compared to those from higher socio-professional categories (+5 pts), the highest income group (+3.2 pts), or experiencing food security (+8 pts), respectively. This was an unexpected finding, given that in France, individuals with low incomes and/or experiencing food insecurity generally have lower diet quality (32), One potential explanation is the higher proportion of “RTECs” breakfast among these groups (Supplementary Table S2). Indeed, RTECs are an affordable source of low-cost nutrients (27), enabling low-income families to access it (27, 30). Many studies on diet quality consider the daily dietary intake, whereas the present study focuses solely on breakfast. Consequently, another explanation could be that, in low income or food insecure households, other meals and snacks contribute to a lower overall nutritional quality, while breakfast may be of higher nutritional quality. On the other hand, children from households where the reference person was from low socio-professional categories were associated with a lower average BQS (−2.5 pts) than those from higher socio-professional categories. In this respect, the BQS value does align with socio-professional level (“low,” “medium” or high”), confirming the socioeconomic gradient (33) typically observed in the overall diet quality of French children (34, 35). In 2019, the French government launched a program to distribute “balanced” breakfasts at school (36) but since our consumption data are from 2014–2015, it does not explain the higher BQS in lower income and food insecure households. Nevertheless, the current food inflationary environment is likely reinforcing existing inequalities in access to better diet quality (37).

Exploring the determinants of breakfast nutritional quality can be more intricate than what categorization and models can fully capture. Further investigations are necessary to comprehensively understand the impact of socio-demographic factors on breakfast nutritional quality. The current BQS is well-adapted tool to analyze breakfast quality at the population level. Individual use may require adjustments to the algorithm, such as implementing stricter penalties for certain nutrients like saturated fats, sodium and free sugars. In the context of an application such as the Breakfast Calculator developed alongside the BQS (20), there may arise a necessity to adjust the algorithm. Our study has a number of limitations. Firstly, while the intake data is the latest available, it dates back to 2015, Since then, there may have been improvements in the nutritional composition of some foods such as RTECs, particularly regarding sugars and fiber content with the incorporation of whole grains. The French public health policy, through Nutri-Score, has encouraged the food industry to reformulate their products (38). Secondly, our analysis is based on observed breakfasts in France, where breakfast patterns predominantly consist of sweet products. This may not be representative of breakfast habits in other countries, such as the UK, where savory items are also common. However, since the BQS is based on IBRI recommendations developed for countries with predominantly Western diet patterns, it theoretically could be applied to children from countries other than France.

In conclusion, the BQS has been shown to be a valuable tool for evaluating the nutritional quality of children’s breakfasts. Breakfast consisting of RTECs and milk emerged as the breakfast type with the highest BQS, while those with biscuits and French viennoiseries and refined breads had the lowest BQSs. There were contradictory disparities in breakfast quality based on socio-economic status which warrant further investigation.

## Data availability statement

Publicly available datasets were analyzed in this study. This data can be found at: https://www.data.gouv.fr/fr/datasets/donnees-de-consommations-et-habitudes-alimentaires-de-letude-inca-3/.

## Ethics statement

The studies involving humans were approved by Comité consultatif sur le traitement de l’information en matière de recherche dans le domaine de la santé. The studies were conducted in accordance with the local legislation and institutional requirements. Written informed consent for participation was not required from the participants or the participants’ legal guardians/next of kin because verbal informed consent was obtained from all subjects.

## Author contributions

RP: Conceptualization, Data curation, Formal analysis, Writing – original draft. SH: Conceptualization, Funding acquisition, Supervision, Writing – review & editing. MM: Conceptualization, Formal analysis, Methodology, Supervision, Writing – review & editing.
