# Supplementary material for: Evaluation of the nutritional quality of French children breakfasts according to the Breakfast Quality Score (BQS)
Source: Front Nutr. 2024 Jul 3;11:1430831. doi: 10.3389/fnut.2024.1430831 (PMC11252056; doi:10.3389/fnut.2024.1430831)
Supplement: Supplementary file 1 [file Data_Sheet_1.docx]

Supplementary Material

Evaluation of the nutritional quality of French children breakfasts according to the Breakfast Quality Score

Romane Poinsot^1*^, Sinead Hopkins^2^, Matthieu Maillot^1^

^1^ MS-Nutrition, Marseille, France

^2^ Cereal Partners Worldwide, Lausanne, Switzerland

*** Correspondence:**Corresponding Author
romane.poinsot@ms-nutrition.com

# Supplementary Figures and Tables

For more information on Supplementary Material and for details on the different file types accepted, please see [here](https://www.frontiersin.org/guidelines/author-guidelines#supplementary-material).

## Supplementary Tables

**Supplemental table 1.** Regularity of breakfast consumption for all 4-17 children and by sex and age range, number of participant and weighted percentages.

| Variable | Modality | Frequency of breakfast | n (participant) | % (weighted) |
| --- | --- | --- | --- | --- |
| All | All  n=1,448 | 0 | 27 | 2.97 |
|  |  | 1 over 3 recalls | 35 | 2.97 |
|  |  | 2 over 3 recalls | 140 | 10.9 |
|  |  | As many as recalls | 1,246 | 83.2 |
| Sex  (chi2  p-value  =0.114) | Male  n=738 | 0 | 18 | 4.54 |
|  |  | 1 over 3 recalls | 21 | 3.32 |
|  |  | 2 over 3 recalls | 76 | 12.0 |
|  |  | As many as recalls | 623 | 80.2 |
|  | Female  n=710 | 0 | 9 | 1.24 |
|  |  | 1 over 3 recalls | 14 | 2.59 |
|  |  | 2 over 3 recalls | 64 | 9.68 |
|  |  | As many as recalls | 623 | 86.5 |
| Age range  (chi2  p-value  <0.001) | 4-6  n=326 | 0 | 2 | 0.56 |
|  |  | 1 over 3 recalls | 4 | 2.44 |
|  |  | 2 over 3 recalls | 17 | 5.70 |
|  |  | As many as recalls | 303 | 91.3 |
|  | 7-10  n=448 | 0 | 1 | 0.18 |
|  |  | 1 over 3 recalls | 4 | 1.10 |
|  |  | 2 over 3 recalls | 33 | 10.9 |
|  |  | As many as recalls | 410 | 87.8 |
|  | 11-13  n=420 | 0 | 10 | 4.74 |
|  |  | 1 over 3 recalls | 11 | 3.16 |
|  |  | 2 over 3 recalls | 44 | 12.2 |
|  |  | As many as recalls | 355 | 79.9 |
|  | 14-17  n=254 | 0 | 14 | 10.2 |
|  |  | 1 over 3 recalls | 16 | 7.72 |
|  |  | 2 over 3 recalls | 46 | 17.5 |
|  |  | As many as recalls | 178 | 64.6 |

Supplemental table 2. Distribution of breakfast categories by socio-demographic modalities

| Variable | Modality | All breakfasts | Biscuits & viennoiseries  N=1,558 | Refined breads  N=1,078 | Wholegrains bread  N=127 | RTECs  N=912 | Non-cereal  N=340 |
| --- | --- | --- | --- | --- | --- | --- | --- |
| Socio-professional category  p-val=0.023 | Low | 36.7 | 38.4 | 34.0 | 19.5 | 37.2 | 41.4 |
|  | Medium | 32.6 | 32.7 | 31.2 | 40.0 | 33.0 | 31.7 |
|  | High | 26.4 | 25.7 | 31.4 | 27.8 | 24.2 | 21.5 |
|  | Inactive | 4.29 | 3.16 | 3.32 | 12.7 | 5.64 | 5.36 |
| Household monthly income per consumption unit  p-val=0.008 | <900 € | 21.9 | 24.0 | 17.6 | 20.1 | 23.2 | 21.0 |
|  | [900-1 340[ € | 27.7 | 26.9 | 23.0 | 18.6 | 30.6 | 39.5 |
|  | [1 340-1 850[ € | 29.3 | 28.4 | 34.3 | 47.0 | 27.2 | 18.9 |
|  | >=1 850 € | 14.5 | 14.4 | 17.3 | 11.3 | 12.9 | 13.2 |
|  | No answer | 6.59 | 6.23 | 7.79 | 3.11 | 6.17 | 7.37 |
| Study level  p-val=0.032 | Primary and middle schools | 39.2 | 39.3 | 36.6 | 31.5 | 42.6 | 39.4 |
|  | Highschool | 18.7 | 20.6 | 16.5 | 6.0 | 16.8 | 26.6 |
|  | Undergraduate studies | 20.2 | 20.0 | 22.3 | 33.7 | 18.7 | 14.7 |
|  | Graduate studies | 21.8 | 20.1 | 24.6 | 28.8 | 21.9 | 19.3 |
| Sex  p-val=0.281 | Male | 51.4 | 52.7 | 50.1 | 53.0 | 53.7 | 42.4 |
|  | Female | 48.6 | 47.3 | 49.9 | 47.0 | 46.3 | 57.6 |
| Age range p-val<0.001 | 4-6 years old | 27.2 | 28.7 | 21.5 | 32.2 | 20.2 | 54.2 |
|  | 7-10 years old | 34.2 | 34.8 | 37.6 | 27.3 | 34.8 | 22.3 |
|  | 11-14 years old | 25.6 | 26.3 | 26.3 | 18.4 | 29.0 | 13.6 |
|  | 15-17 years old | 13.0 | 10.2 | 14.6 | 22.1 | 16.0 | 9.87 |
| Food insecurity level  p-val=0.115 | Food Security | 91.6 | 91.8 | 93.1 | 94.2 | 88.2 | 95.4 |
|  | Moderate food insecurity | 6.21 | 6.17 | 5.15 | 2.98 | 9.21 | 2.25 |
|  | Severe food insecurity | 2.15 | 2.05 | 1.73 | 2.79 | 2.58 | 2.38 |
| Physical activity level  p-val=0.020 | Low | 37.4 | 36.4 | 37.8 | 34.7 | 40.2 | 34.0 |
|  | Medium | 37.6 | 38.8 | 37.3 | 24.0 | 33.4 | 50.4 |
|  | High | 25.0 | 24.8 | 24.8 | 41.3 | 26.5 | 15.6 |
| Screen time  p-val=0.98 | Half an hour or less | 76.0 | 76.1 | 74.9 | 76.8 | 76.9 | 75.7 |
|  | Between 0.5 and 1 hour | 19.4 | 19.9 | 20.4 | 18.5 | 17.6 | 19.3 |
|  | More than 1 hour | 4.60 | 3.91 | 4.68 | 4.72 | 5.49 | 5.03 |
| BMI classes  p-val=0.013 | Thinness | 12.3 | 9.63 | 11.0 | 16.9 | 13.7 | 22.8 |
|  | Normal | 74.6 | 77.2 | 73.9 | 65.9 | 74.8 | 67.7 |
|  | Overweight and obesity | 13.1 | 13.2 | 15.1 | 17.2 | 11.5 | 9.56 |


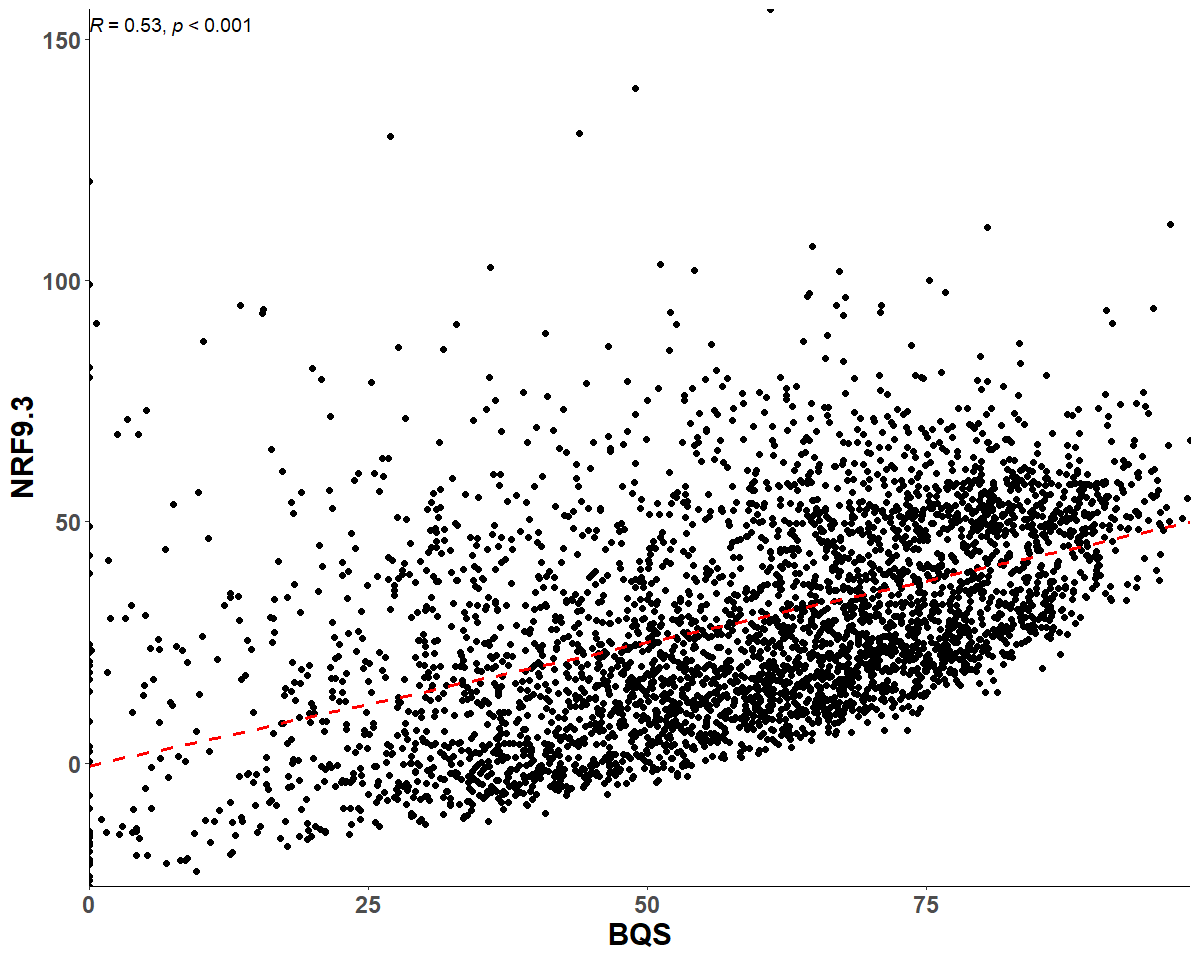


Supplemental Figure 1. Breakfast Quality Score (BQS) against NRF 9.3
